# Macroplastique for women with stress urinary incontinence secondary to intrinsic sphincter deficiency

**DOI:** 10.1590/S1677-5538.IBJU.2019.0070

**Published:** 2019-01-29

**Authors:** Timothy F. Carroll, Alana Christie, Melissa Foreman, Gaurav Khatri, Philippe E. Zimmern

**Affiliations:** 1 University of Texas Southwestern Medical Center TX USA University of Texas Southwestern Medical Center, TX, USA

**Keywords:** Urinary Incontinence, Urinary Sphincter, Artificial, Women

## Abstract

**Objective:**

To evaluate the subjective and objective outcomes of Macroplastique® (MPQ) in women with stress urinary incontinence (SUI) secondary to intrinsic sphincter deficiency (ISD).

**Materials and Methods:**

Following Institutional Review Board (IRB) approval, charts of non-neurogenic women with SUI secondary to ISD who underwent MPQ injection and had 6 months minimum follow-up were reviewed from a prospectively maintained database. Patients were divided into 3 groups: Naïve (Group I), Prior Anti-Incontinence Surgery (Group II), and combined Prior Bulking Agent and Anti-Incontinence Surgery (Group III). Data collected included SUI self-report, Urogenital Distress Inventory (UDI-6) Question 3, and VAS Quality of Life (QoL) Questionnaire scores at baseline and in follow-up. Three-dimensional ultrasound (3DUS) evaluated volume/configuration of MPQ. Success was defined after the last MPQ injection as a UDI-6 Question 3 score of 0 (dry) or 1, and no reoperation for SUI.

**Results:**

From 2011-2017, 106 of 142 women met study criteria. At a median follow-up of 20 months (mean=26 months; range: 6-71), success rate was 41% for Group I, 40% for Group II, and 65% for Group III (p = 0.22). QoL scores were significantly improved over baseline in all groups. There was no significant difference in clinical outcome between the asymmetrical and symmetrical group on 3DUS. The completely dry rate was highest in Group III at 29%, compared to 4% for Group I and 15% for Group II (p = 0.05).

**Conclusion:**

Macroplastique® improved subjective and objective outcome measures for SUI secondary to ISD as both a primary and secondary treatment option in women.

## INTRODUCTION

Macroplastique® (Cogentix Medical, Orangeburg, New York, USA) (MPQ) is a bulking agent used in the treatment of stress urinary incontinence (SUI) secondary to intrinsic sphincter deficiency (ISD). MPQ has been available since 1991 in Europe, but has only been used at our institution since 2011 when Collagen (Contigen™) was no longer available. The material is composed of both the resorbed carrier, polyvinylpyrrolidone, and the permanent silicone-like elastomer, polydimethylsiloxane beads.

While Collagen was generally regarded as a safe and efficacious injectable, less literature is available on MPQ outcomes and the factors that may predict its long-term success. In a randomized controlled trial comparing MPQ to Collagen, Ghoniem and colleagues reported that MPQ injection resulted in a statistically significant 12% increase in dry/cure rate over the group receiving Collagen ( 1 ). A few series have reported MPQ success rates between 40-85% over more than 2 years using a variety of outcome measures including patient self-report, urodynamics, and Stamey grades ( 2 , 3 ). Our group has only examined the short-term outcome of MPQ and reported a success rate of 75% in 59 women followed for a mean of 9 months ( 4 ).

In this report, as we did for Collagen in the past ( 4 - 6 ), we examined our longer term experience with MPQ using subjective and objective (three-dimensional ultrasound (3DUS)) outcome criteria as well as studied factors that could predict success over time in order to better counsel patients with bothersome incontinence interested in a minimally invasive approach.

## MATERIALS AND METHODS

After Institutional Review Board approval, a review from a prospectively maintained database of women treated with MPQ injection was performed. Included in this study were women with bothersome stress urinary incontinence due to ISD and with a radiologically proven well-supported urethra. Excluded were those with follow-up <6 months, a neurogenic bladder, or an indwelling suprapubic tube.

The diagnosis of ISD was based on several criteria including: a positive supine stress test, a well-supported urethra as confirmed by standing cystourethrogram revealing minimal difference in urethral angle between rest and straining ( 7 ), and Valsalva leak point pressure (VLPP) obtained during urodynamic studies performed according to the Urinary Incontinence Treatment Network protocol ( 8 ). Similar to prior studies on Collagen, we did not use a specific cutoff for VLPP to diagnose ISD, although a higher VLPP may indicate a less severe form of ISD ( 9 , 10 ).

**Figure 1:**
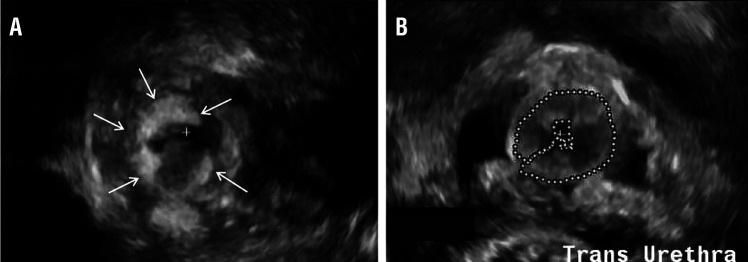
(+)=Urethral lumen; 3DUS=Three-dimensional ultrasound. 3DUS reveals asymmetric MPQ configuration (A) compared to a symmetrical “horseshoe configuration” (B). MPQ is indicated by either arrows (A) or as outlined (B).

The same urologist (PZ) performed MPQ injection on an outpatient basis under light anesthesia (monitored anesthesia care or laryngeal mask) using a 21F Wolf cystoscope with a 30˚ lens. Following our experience with Collagen, injections were made transurethrally at the 3 and 9 o’clock positions at the mid-urethral level. MPQ tracked around the urethra both superiorly and inferiorly as confirmed by subsequent 3DUS ( Figure-1 ). A total of 5mL MPQ was generally injected, typically with 2.5mL at each injection site. Patients who failed their voiding trial (complete retention or voiding less than half of bladder capacity) were discharged home with a small urethral catheter (12Fr-14Fr) for 24 hours.

As in our prior early study on MPQ, women were grouped into 3 different categories: Naïve (Group I), previous anti-incontinence surgery (Group II), or prior anti-incontinence procedure and bulking agent (Group III) ( 4 ). Prior anti-incontinence surgeries included: bladder neck suspension, autologous sling, removal of a prior synthetic sling/urethrolysis, and/or associated prolapse repair addressing the anterior and apical compartments (e.g. suspensions involving the anterior wall, sacrocolpopexy, and apical suspension).

Subjective outcomes were evaluated through patient self-report of improvement, question 3 of the validated Urogenital Distress Inventory-Short From (UDI-6) (“Do you experience, and if so, how much are you bothered by leakage related to physical activity, coughing, or sneezing?”; 0=not at all, 1=slightly, 2=moderately, 3=greatly), and the VAS Quality of Life Questionnaire (QoL) (“With regards to the impact your bladder condition has on your life, how would you describe your current quality of life?” with answers ranging from 0=pleased to 10=terrible) ( 11 ). These subjective outcome measures were recorded both at baseline (pre-injection) and at each follow-up visit. Women who did not return after 6 months for follow-up were administered the same questionnaires (self-report, UDI-6, QoL) over a structured telephone interview by a neutral investigator not involved in the care of these patients.

Three dimensional ultrasound (3DUS) evaluations were performed at 6-8 weeks postoperatively and at 1-2 year intervals thereafter. These measurements were made using the Philips IU22 ultrasound system (Philips Healthcare, Bothell, WA) with endovaginal 3D 9-3V end-fire mechanical probe with automatic 3-D multi-planar image acquisition. 3DUS evaluations were performed with the patient in the dorsal lithotomy position with a moderately distended bladder. MPQ was easy to identify as it is very echogenic. Volume calculations were made using the stacked contour method. As in our prior study on Collagen injection ( 12 ), the radiology report contained information on whether the MPQ configuration was symmetrical (circumferential/horseshoe shape of MPQ around the urethra) or asymmetrical (MPQ lacking in one area) ( Figure-1 ). The ultrasound examination and volume/configuration measurements were originally obtained by an ultrasound technician at the time of evaluation, then re-measured by the same senior sonographer (MF) for the whole study. The images were then reviewed by a radiologist for final interpretation. The sonographer and radiologist were unaware of patient outcomes. In addition, our senior sonographer (MF) randomly chose to re-read 10 studies to determine her intra-rater reliability, and had a Pearson’s correlation at 0.999 (95% CI 0.993-0.999).

The primary outcome of the study, “success”, was defined after the last MPQ injection as a UDI-6 Question 3 score of 0 or 1 and no surgical reoperation for SUI. A repeat MPQ injection was not considered failure. Secondary outcome variables included VAS QoL score as well as patient self-report of improvement. Additionally, patients were considered “dry/cure” with a self-report of 100% improvement or a UDI-Question 3 score of 0 were observed.

## STATISTICAL METHODS

Descriptive statistics were provided as medians, means, standard deviations, and ranges for continuous factors, and frequencies and percentages for categorical factors. We used the Chi-square test to determine if categorical factors were significantly associated with prior incontinence treatment history or with success versus failure of the MPQ, and the Student t-test for continuous factors. Differences in questionnaire results were compared between pre-MPQ and last follow-up using the paired t-test. All analyses were completed at the 0.05 significance level using SAS 9.4 (SAS Institute Inc., Cary, NC).

## RESULTS

From 2011-2017, 106 of 142 women met study criteria, including 16 who completed phone interviews. Of the 36 excluded, 30 were lost to follow-up, 5 had a neurogenic bladder, and 1 had a suprapubic tube. There were no serious adverse events in our series, including no systemic complications observed.

Mean age was highest in Group III (73 years), while BMI was not significantly different amongst the groups. The majority of patients in each group only had 1 injection, while the remainder had 2 or more within the study period at a mean interval time of 9 months between first and second injection. Pre-injection urodynamic findings were not different amongst the groups with the exception of Qmax, which was highest in Group I (19.6mL/sec) (p=0.03).

At a median follow-up of 20 months (mean=26 months; range: 6-71 months), the success rate was 45% with minimal differences between the 3 groups (41% for Group I, 40% for Group II, and 65% for Group III (p=0.22)). Group III had a significantly higher success rate after the first MPQ injection (59%) compared to Group I (37%) and Group II (25%) (p=0.03). Of 26 women with follow-up over 3 years, 58% met success criteria as did 75% of 8 women with follow-up of 5 years or more. The completely dry/cure rate was 4% for Group I, 15% for Group II, and 29% for Group III (p=05) ( Table-1 ). Improvements in UDI-6 Question 3 scores were not significantly different across groups.

**Table 1:** Patient characteristics by prior incontinence treatment history.

	Naive (n=28)	Prior Surgery (n=61)	Prior Collagen± Surgery (n=17)	p
Age, years (range)	65.4±8.3 (45-81)	63.4±11.1 (37-92)	72.9±7.9 (59-88)	0.0036
BMI	25.8±5.3	28.8±6.7	27.7±5.8	0.1202
Gravida	2.5±1.3	2.9±1.3	2.7±1.4	0.3514
Parity	1.9±1	2.5±1.2	2.6±1.4	0.0618
**Hysterectomy**				
No	6 (21%)	10 (16%)	3 (18%)	0.8312
Yes	22 (79%)	51 (84%)	14 (82%)	
**HRT**				
No	13 (46%)	33 (54%)	9 (53%)	0.8296
Yes	15 (54%)	28 (46%)	8 (47%)	
VLPP, cm H _2_ O (range)	62.6±32.1 (20-160)	61.7±30.9 (17-140)	46.6±27.0 (16-88)	0.4179
Qmax, mL/sec (range)	19.6±8.9 (4.2-41)	16.1±8.5 (5.8-50)	12.6±6.0 (3-24)	0.0296
Years between 1 ^st^ and 2 ^nd^ MPQ	0.5±0.3	0.8±0.6	1.0±0.7	0.3072
Retention after 1 ^st^ MPQ	10 (36%)	25 (41%)	10 (59%)	0.3243
Retention after 2 ^nd^ MPQ	2 (33%)	12 (55%)	1 (25%)	0.5014
Retention after final MPQ	10 (36%)	29 (48%)	9 (53%)	0.4672
Completely dry	1 (4%)	9 (15%)	5 (29%)	0.0517
Success after first MPQ	10 (37%)	14 (25%)	10 (59%)	0.0331
Success after final MPQ	11 (41%)	23 (40%)	11 (65%)	0.2229

**BMI=** Body Mass Index; **HRT=** Hormone Replacement Therapy; **VLPP=** Valsalva Leak Point Pressure; **Qmax=** Maximum urinary flow rate.

Patient characteristics according to the respective group based on prior incontinence treatment history. Age and Qmax were significantly different at baseline. Success after first MPQ injection and completely dry rate after final MPQ injection are highest in Group III (p=0.03 and 0.05, respectively).

Table-2 details an analysis of factors that could potentially predict success. Overall, transient retention rate was 45% after the last MPQ injection. Retention after final MPQ and the number of MPQ injections were near statistical significance in predicting success at last follow-up (p=0.07). Pre-operative urodynamic values did not show a significant relationship with MPQ success, including VLPP, which was also not associated with UDI-6 Question 3 scores when ranked from 0-3 (p=0.54). The pre-injection UDI-6 Question 3 scores were not different between the success and failure groups, arguing against the possibility that ISD severity influenced our MPQ outcomes. QoL scores at baseline were significantly lower in the success group. Five patients with missing UDI-6 Question 3 score but all other complete data were separated from this analysis ( Table-2 ).

**Table 2:** Patient characteristics by success after final Macroplastique.

	Failed Macroplastique (n=56)	Successful Macroplastique (n=45)	No UDI-6 Q3, no reoperation (n=5)	p
Age, years (range)	63.4±10.7 (37-85)	68.1±9.9 (46-92)	64.8±9.6 (57-78)	0.1203
BMI	28.5±7.3	26.9±4.8	27.8±5.0	0.8449
Pregnancy	2.8±1.4	2.7±1.2	2.0±1.6	0.5333
Parity	2.4±1.2	2.3±1.2	2.0±1.4	0.9090
**Hysterectomy**				
No	11 (20%)	7 (16%)	1 (20%)	0.9142
Yes	45 (80%)	38 (84%)	4 (80%)	
**HRT**				
No	27 (48%)	25 (56%)	3 (60%)	0.7146
Yes	29 (52%)	20 (44%)	2 (40%)	
UDI6 Q3 (0-3)	2.7±0.5	2.6±0.7	2.3±1.5	0.8973
QoL Score (0-10)	8.3±2.2	7.3±2.5	9.8±0.5	0.0412
Retention after 1 ^st^ MPQ	19 (34%)	24 (53%)	2 (40%)	0.1383
Retention after 2 ^nd^ MPQ	10 (43%)	5 (56%)	-	0.6989
Retention after final MPQ	20 (36%)	26 (58%)	2 (40%)	0.0796
Volume voided	272.9±155.8	197.6±124.3	281.8±119.0	0.4148
Post-void residual	29.8±70.0	7.8±21.4	11.3±22.5	0.0968
Median VLPP, cm H _2_ O	55	60	40	0.9884
Mean VLPP (range)	60.1±30.2 (17-160)	60.0±31.0 (16-140)	64.0±48.7 (32-120)	0.9790
VLPP <60, cm H _2_ O	23 (55%)	13 (46%)	2 (67%)	0.7174
VLPP ≥60, cm H _2_ O	19 (45%)	15 (54%)	1 (33%)	
Qmax, mL/sec (range)	15.4±8.4 (4.2-50)	17.7±8.7 (3-41)	17.2±8.0 (10-30)	0.3276
Pdet Qmax, cm H _2_ O (range)	15.6±11.6 (0-45)	13.0±9.8 (0-40)	7.6±2.5 (5-10)	0.3407
**Number of injections**				
1	32 (57%)	34 (76%)	5 (100%)	0.0705
2	19 (34%)	11 (24%)	0 (0%)	
3	5 (9%)	0 (0%)	0 (0%)	

**UDI-6 Q3=** UDI-6 Question 3; **QoL=** Quality of Life; **VLPP=** Valsalva Leak Point Pressure; **Qmax=** Maximum urinary flow rate; **Pdet Qmax** =Detrusor pressure at Qmax

Details an analysis of factors predicting success in patients receiving MPQ. Retention after first MPQ injection and number of injections approach significance. Baseline QoL score is highest (worst) in those without UDI-6 Question 3 after MPQ injection but also with no reinjection to indicate failure.

Table-3 illustrates the differences in UDI-6 and QoL scores with each group from pre-injection to last follow-up. A statistically significant improvement was observed with Groups I and II in total UDI-6 score. Groups I and II also reported significant improvement in UDI-6 Question 2 (urgency). Each group experienced significant improvement in UDI-6 Question 3 score (stress). Only Group I experienced significant improvement in UDI-6 Question 5 (ability to empty), although Group II approached significance (p=0.07). QoL scores were significantly improved over baseline in all groups. Of 19 women with low baseline UDI-6 Question 2 scores of 0-1, only 6 reported increases in score over time after MPQ, indicating a low incidence of de novo urgency/urge incontinence symptomatology.

**Table 3:** Changes in UDI-6 and QoL questionnaire responses by prior incontinence treatment history.

	n	Pre-MPQ Mean	Last FU Mean	Mean difference (95% CI)	p
**UDI-6 Total Score (0-36)**					
Naive	17	10.5±4.2	6.6±4.2	-3.4 (-5.5, -1.3)	0.0030
Prior surgery	41	11.9±3.3	8.2±4.7	-3.7 (-5.2, -2.3)	<0.0001
Prior collagen±surgery	10	8.9±2.5	6.3±4.5	-2.0 (-6.2, 2.2)	0.3048
**UDI-6 Q2 UUI (0-3)**					
Naive	20	2.0±1.2	1.1±1.1	-0.6 (-1.2, 0.01)	0.0535
Prior surgery	48	2.4±0.9	1.8±1.1	-0.6 (-0.9, -0.3)	0.0008
Prior collagen±surgery	13	2.4±0.9	1.6±1.2	-0.3 (-1.1, 0.5)	0.4156
**UDI-6 Q3 SUI (0-3)**					
Naive	22	2.8±0.4	1.9±1.0	-0.8 (-1.3, -0.4)	0.0010
Prior surgery	49	2.6±0.7	1.7±1.0	-0.9 (-1.2, -0.6)	<0.0001
Prior collagen±surgery	14	2.4±0.6	1.2±1.0	-0.9 (-1.6, -0.3)	0.0094
**UDI-6 Q5 Empty (0-3)**					
Naive	20	0.8±1.2	0.3±0.7	-0.5 (-0.9, 0.02)	0.0583
Prior surgery	45	1.0±1.1	0.7±1.0	-0.3 (-0.6, 0.03)	0.0702
Prior collagen±surgery	14	0.6±0.9	0.7±0.9	0.2 (-0.5, 0.9)	0.5328
**Quality of Life Score (0-10)**					
Naive	14	7.1±2.5	4.4±3.2	-2.1 (-3.8, -0.3)	0.0256
Prior surgery	45	8.6±2.0	4.6±3.4	-3.9 (-4.9, -2.9)	<0.0001
Prior collagen±surgery	12	6.5±2.6	3.9±2.8	-2.0 (-4.1, 0.1)	0.0580

**UDI-6** =Urogenital Distress Inventory; **QoL=** Quality of Life questionnaire; **FU=** Follow-Up; **UUI=** Urge Urinary Incontinence; **Q=** Question; **SUI=** Stress Urinary Incontinence

Women with a symmetric distribution of MPQ at last follow-up had higher rates of both completely dry/cure (18% of symmetric vs. 6% of asymmetric) and success (48% of symmetric vs. 33% of asymmetric), although these differences were not statistically significant (p=0.22 and 0.19, respectively). Symmetry was not associated with post-injection UDI-6 Question 3 or QoL scores. After controlling for time since last MPQ injection and total number of injections, no association between volume and success was found (p=0.83).

In the “prior surgery” group (n=61), 13 had prior prolapse repair alone, including sacrocolpopexy ( 3 ), anterior vaginal wall suspension ( 4 ) and anterior-posterior repair ( 4 ). Of the 48 who had prior incontinence surgery, 17 had an autologous fascial sling and 31 a sub-urethral synthetic sling release. No difference was noted in retention rate immediately post-MPQ injection (42% in both groups), or in success rate (41% after sling placement, 39% after sling release).

**Figure 2:**
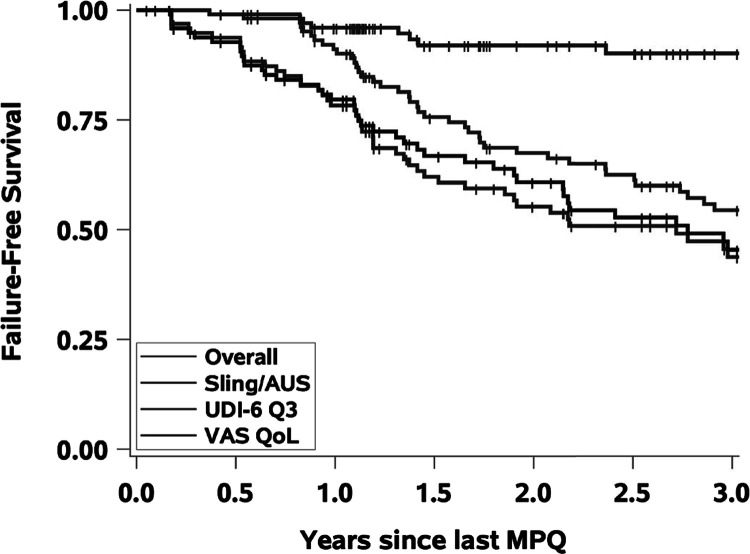
The red line represents the 10 patients who proceeded to Sling/AUS after last MPQ injection. The remaining three lines represent the entire cohort.

Among the 56 failures (defined as those that did not meet criteria for “success”), 32 failed after 1 injection, 20 after 2 injections, and 4 after 3 injections. Ten of fifty-six failures proceeded to autologous sling ( 8 ) or artificial urinary sphincter (AUS) placement ( 2 ) at a mean follow-up interval of 8 months after the last MPQ injection. Four of these 10 patients proceeded to autologous sling after the first injection, while the remaining six patients had two or three injections before sling or AUS insertion. Kaplan-Meier survival analysis based on failure criteria revealed a gradual decline in questionnaire scores over years and a latency in time to receiving fascial sling/AUS after injection ( Figure-2 ).

## DISCUSSION

Our study examined the outcome of MPQ injection using subjective and objective outcome measures with a success rate of 45% after the last MPQ injection at a median of 20 months, and with minor variance amongst our 3 MPQ indication subgroups. In a subset of women with longer follow-up at 3 and 5 years, the success rates remained comparable.

Success rates for MPQ at different time periods are variable in the literature ( Table-4 ) and differ based on definition of success and the tools to define outcomes. A meta-analysis by Ghoniem and Miller reported a success rate of 64% at 18 months, although the analyzed reports used different methods of determining outcomes ( 13 ). Longer-term findings have been reported by Tamanini and colleagues who used post-operative VLPP to establish a cure/improvement rate of 73% in 15 women followed for 60 months ( 3 ). Zullo and colleagues reported on a series of 61 women with a success rate of 57% at a follow-up point of 60 months ( 14 ), with success defined as cure or improvement in voiding symptoms (measured with voiding diary and cough stress test).

**Table 4:** Success Rates in the Literature.

Study	Study Design	Method of Determining Success		N	Follow-Up (months)		Dry Rate (%)		Improvement Rate (%)	
Ghoniem et al. (1)	Randomized, single-blind	Stamey Grade		122	12		36.9		61.5	
Ghoniem et al. (2)	Case Series	Stamey Grade		67	24		67		84	
Ghoniem and Miller (13)	Meta-Analysis	Variable		Variable	>18		36		64	
Harriss et al. (20)	Case Series	Self-Report		40	36		40		18	
Maher et al. (21)	RCT*	Self-Report		22	12		n/a		60	
Plotti et al. (22)	Case Series	Voiding Diary/Stress Test		24	12		42		42	
Radley et al. (18)	Case Series	Self-Report		56	19		20		59	
Rosenfeld et al. (4)	Case Series	Questionnaires/Self-Report		59	9		19		75	
Serati et al. (19)	Prospective Cohort	Questionnaires/Stress Test		85	36		47		49	
Tamanini et al. (3)	Prospective Cohort	Urodynamic Testing		15	60		40		33	
Zullo et al. (14)	Prospective Cohort	Voiding Diary/Stress Test		61	60		18		39	

*RCT= Randomized Controlled Trial

Group III had the highest completely dry/cure rate (29%) and overall success rate (65%) after final MPQ injection, despite the latter value not reaching statistical significance. This experience parallels that of Gumus and colleagues, whose report compared MPQ outcomes using I-QOL, IIQ-7, and UDI-6 questionnaires in 35 women with and without history of anti-incontinence procedures ( 15 ). This group also found that at a median follow-up of 58 months, women receiving MPQ after failed prior SUI surgery were more satisfied with the outcome of MPQ injection.

Factors predicting success after MPQ injection were studied. First, a repeat MPQ injection led to a nearly 25% increase in success over time. Second, transient post-operative retention, although not statistically significant, was considerably higher in the success group (58%) than in the failure group (36%). Post-operative urinary retention is reportedly found in approximately 6-32% of women receiving MPQ injection ( 13 ), while the rate in our series was 45% after final injection. Third, we analyzed volume and configuration of MPQ by 3DUS, a technology employed in different specialties that is well suited to image the vaginal space ( 16 , 17 ). However, its use as a follow-up tool after MPQ injection has only recently been described and therefore, its role as an outcome predictor has not been fully evaluated yet. A prior study of Collagen injection at our institution in 46 women with a mean follow-up of 14 months who were evaluated with 3DUS 4-12 weeks after injection found a correlation between a positive clinical outcome and a symmetrical/circumferential configuration of Collagen ( 12 ). These results have since been corroborated by Radley and colleagues who reported that in 9 women followed for 19 months after MPQ injection, a good clinical outcome was seen with MPQ completely surrounding the urethra compared to a poor outcome in 3 women with incomplete urethral encirclement ( 18 ).

Although not studied in our series, Serati and colleagues found that increased surgeon’s skill and lack of prior radical pelvic surgery significantly correlated with success in 85 women injected with MPQ and followed for 3 years ( 19 ). Notable differences with the present study include the lack of 3DUS data as well as the decision to not offer reinjection should patients require further anti-incontinence treatment after the first MPQ injection.

Strengths of our study included several ISD groups based on prior SUI treatment history, a relatively large sample size, and a mid-term follow-up including SUI self-reporting and validated questionnaires. In addition, this study utilized 3DUS as an objective outcome measure. Our efforts to reach all participants over the phone to optimize our long-term results and limit our loss to follow-up data were met with limitations, as in any real-life practice study. Slight differences across our three groups at baseline could also have had an impact as they varied in age and Q-max.

## CONCLUSIONS

MPQ resulted in improved UDI-6 scores, QoL scores, and self-reported continence as both a primary and secondary treatment option in women with SUI secondary to ISD. MPQ may be particularly valuable in those with more extensive prior anti-incontinence treatments. Factors that could predict success such as repeat MPQ injection, immediate post-MPQ retention, and injection configuration/volume by 3DUS will have to be tested over longer term follow-up.
